# Intracellular oligonucleotide delivery using the cell penetrating peptide Xentry

**DOI:** 10.1038/s41598-018-29556-7

**Published:** 2018-07-26

**Authors:** Frazer P. Coutinho, Colin R. Green, Ilva D. Rupenthal

**Affiliations:** 1 Buchanan Ocular Therapeutics Unit, Department of Ophthalmology and the New Zealand National Eye Centre, University of Auckland, Auckland, New Zealand; 2 Department of Ophthalmology and the New Zealand National Eye Centre, University of Auckland, Auckland, New Zealand

## Abstract

The current study investigated the use of two cationic peptides, Xentry-KALA (XK) and Xentry-Protamine (XP), for intracellular delivery of Connexin43 antisense oligonucleotides (Cx43AsODN). The charge and size of Cx43AsODN:XK and Cx43AsODN:XP complexes was determined by Zetasizer analysis. The earliest positive zeta potential reading was obtained at a 1:2 and 1:1.2 charge ratio of Cx43AsODN:XK and Cx43AsODN:XP respectively, with Cx43AsODN:XK resulting in overall larger complexes than Cx43AsODN:XP. Gel shift mobility assays revealed complete complex formation at a 1:2.5 and 1:2.2 charge ratio of Cx43AsODN:XK and Cx43AsODN:XP, respectively. Cellular uptake studies were carried out in ARPE-19 cells. While both complexes were able to enter the cells, Cx43AsODN:XK uptake appeared punctate and circular indicative of endosomal containment. Cx43AsODN:XP uptake, in contrast, resulted in diffuse appearance inside the cell suggesting endosomal escape of the cargo. Finally, western blot analysis confirmed that Cx43AsODN:XP was able to knockdown Cx43 expression in these cells under normal and hypoxic conditions.

## Introduction

Connexins are a family of proteins responsible for the formation of communication channels between cells called gap junctions^1^. Gap junction communication is essential for cell survival, repair and growth processes^2,3^. When six connexin subunits come together they are able to form a membrane pore called a hemichannel. The docking of hemichannels from neighbouring cells creates a gap junction, allowing the passage of molecules between cells^4,5^. There are 21 different connexin proteins expressed in humans and these are named according to their molecular weight. The most studied and well described connexin is the 43 kDa protein, Connexin43 (Cx43)^4^. Cx43 expression has been shown to increase following injury such as in skin wounds, spinal cord injury, cardiovascular disease and other ischaemic and inflammatory conditions, resulting in cell damage and death^6–11^. This is due to the unregulated opening of hemichannels under pathological conditions which are normally closed under physiological conditions. This creates osmotic and ionic imbalances between the cell and the environment eventually resulting in cell death^12–14^. The elevation of Cx43 expression levels during injury increases the probability of open hemichannels and thus cell death. However, reduced gap junction communication post injury has also been linked to poor tissue recovery^15,16^.

The modulation of Cx43 expression using Connexin43 antisense oligonucleotides (Cx43AsODN) has shown therapeutic efficacy in skin wound and spinal cord injury by reducing inflammation, increasing cell survival and promoting tissue recovery^17–19^. Cx43AsODN is a single DNA strand of 30 deoxynucleotides with an unmodified backbone^8,17^. Cx43 mRNA translation is inhibited by Cx43AsODN thus reducing the production of new Cx43 protein. This does not interfere with already existing Cx43 proteins while the effect is also transient^17^. Therefore, Cx43 expression is kept at low to normal levels during injury while adequate levels for gap junction communication post injury are still maintained. As Cx43AsODN is an unmodified oligonucleotide, efficient delivery is challenging as antisense oligonucleotides are easily broken down in the systemic circulation^20–23^. Thus, most studies have employed topical application of Cx43AsODN incorporated into a thermo-reversible gel, Pluronic F-127, to sites of injury^8^.

The eye is an organ rich in Cx43 and therefore Cx43AsODN has therapeutic potential in ocular inflammatory diseases such as corneal surface wounds^24–27^. Cx43AsODN has been shown to reduce Cx43 expression in *in vitro*, *in vivo* and *ex vivo* rat corneal injury models which promoted epithelial recovery^8,28^. The topical application of Cx43AsODN has also been efficacious in the treatment of corneal burns (chemical and thermal) in humans treated on a compassionate use basis, reducing inflammation and promoting corneal reepithelialisation^24^. Finally, Cx43AsODN has also shown therapeutic efficacy in reducing inflammation in optic nerve ischaemia in an organotypic culture model^29^; however, efficient cellular uptake remains a challenge.

Cell penetrating peptides (CPPs) have been used extensively for the transport of cargo molecules into cells^30^. CPPs offer increased stability and bioavailability of the cargo inside the cell^31^. Therefore, the use of CPPs to deliver Cx43AsODN could have significant therapeutic advantages. A new class of CPP, Xentry, derived from the X-protein of the hepatitis B virus has previously been used to successfully transport large molecules such as peptides, antibodies as well as smaller molecules such as siRNA into cells with high efficiency^32,33^. An added advantage of Xentry is that it binds to cell surface expressed Syndecan-4 to initiate clathrin-mediated endocytosis^33^. Syndecan-4 is not expressed by blood cells therefore allowing for systemic delivery of the cargo as the complex would not be sequestered by the blood circulation^33^. A common method of complexing negatively charged nucleotides to positively charged peptides is by non-covalent electrostatic interactions^34–36^. However, Xentry itself only carries one positively charged amino acid, thus fusion with an additional positively charged peptide is required to achieve efficient Cx43AsODN complexation (charge of −30). KALA is a lysine rich amphipathic peptide, able to efficiently bind and condense DNA, as well as induce endosomal membrane leakage thus improving transfection^35^. The fusion of Xentry to KALA results in Xentry-KALA (XK) with an overall charge of +6. XK has previously been shown to successfully transport siRNA into melanoma cells^32^. Protamine is an arginine rich peptide known to condense DNA and increase resistance to enzymatic degradation^36^. The fusion of Xentry to protamine results in Xentry-Protamine (XP) with an overall charge of +13. This study aimed to investigate the ability of these two cationic Xentry peptides, XK and XP, to transport Cx43AsODN across the cell membrane and knockdown Cx43 protein levels post cellular uptake.

## Materials and Methods

### Cell culture and maintenance

Unless otherwise mentioned, all cell culture reagents were purchased from Gibco® or Invitrogen™ (Thermo Fisher Scientific). ARPE-19 cells are from a human retinal pigment epithelium cell line purchased from American Type Culture Collection (ATCC), and were maintained in T25 flasks in DMEM/F12 (DMEM/F-12, GlutaMAX^TM^ medium containing 10% heat inactivated foetal bovine serum (FBS), and 1% Antibiotic-Antimicotic) at 37 °C with 5% CO_2_. Cells were grown to 70–80% confluence and maintained at less than thirty passages. For experiments, cells were harvested with TrypLE™ Express, centrifuged at 1500 RPM for 7 min, re-suspended in culture medium, and counted using trypan blue and the Neubauer haemocytometer. The required concentration of cells was plated and incubated at 37 °C with 5% CO_2_ overnight to allow for cell attachment.

### Xentry peptides and Cx43AsODN

Xentry-KALA (XK) ((biotin)-lclrpvgggweaklakalakalakhlakalakalkacea, MW: 4211.55, 79.37% purity, +6 charges) and Xentry-Protamine (XP) ((biotin)-lclrpvggrsqsrsryyrqrqrsrrrrrrs, MW: 4060.74, 83.42% purity, +13 charges) were purchased from Peptide 2.0 Inc. Connexin43 antisense oligonucleotide (Cx43AsODN) (5′-GTA ATT GCG GCA GGA GGA ATT GTT TCT GTC-3′) (−30 charges) was purchased from Agilent Technologies while Cx43AsODN-Cy3 ((Cy3)-GTA ATT GCG GCA GGA GGA ATT GTT TCT GTC) used to visualise uptake was purchased from Sigma-Aldrich.

### Zeta potential and size measurements of complexes

Zeta potential as well as size measurements were carried out using a Malvern Zetasizer (Malvern Instruments). Cx43AsODN at a concentration of 1 µM was combined with either XK at charge ratios ranging from 1:1.25 to 1:2.5 or with XP at charge ratios ranging from 1:1.1 to 1:1.6. Complexes were mixed in ultrapure water. Statistical analysis of complex sizes was carried out using GraphPad Prism7.

### Gel shift mobility assay of complexes

XK and XP peptides were mixed with 300 ng of Cx43AsODN at increasing charge ratios ranging from 1:0 to 1:2.5 and 1:0 to 1:4.8, respectively. Loading dye (50% glycerol/50% water (v/v)), Orange G (BDH Chemicals) was added before loading each sample onto a 1% Ultrapure™ agarose gel (Life Technologies) in Tris-Borate-Ethylenediaminetetraacetic acid (TBE) buffer containing 1:25,000 GelRed™ (Thermo Fisher Scientific) nucleic acid stain. The gel was run at 80 V for 20–30 min and Cx43AsODN migration was visualized using the Gel Doc EZ imaging system (Bio-Rad Laboratories Inc).

### Cellular uptake of complexes

ARPE-19 cells were harvested and seeded in 8-well chamber slides at a density of 2 × 10^5^ cells/ml in DMEM/F12 and incubated overnight at 37 °C and 5% CO_2_. Cx43AsODN:XK and Cx43AsODN:XP complexes at charge ratios of 1:2.5 or 1:2.2 respectively were mixed in DMEM/F12 and incubated at room temperature for 10 min before being applied to cells and incubated for 4 h at 37 °C and 5% CO_2._ Solutions were removed and cells were washed in PBS before fixation with 4% formaldehyde in PBS. Nuclei were counterstained with DAPI and slides were mounted with CitiFluor™ AF-1 and cover slipped. Cells were observed by fluorescent microscopy (Leica DMRA).

### Condensed cell quantification

ARPE-19 cells were harvested and seeded in 8-well chamber slides at a density of 2 × 10^5^ cells/ml in DMEM/F12 and incubated overnight at 37 °C and 5% CO_2_. XP was applied to cells in DMEM/F12 at 0, 1, 5, 10, 15, 20 and 25 µM concentrations and incubated for 24 h at 37 °C and 5% CO_2_. Cells were observed by bright field microscopy and five images per concentration were acquired. Condensed cell bodies with altered morphology were counted using ImageJ and statistical analysis was carried out using GraphPad Prism7.

### Cx43 expression levels via Western Blot

ARPE-19 cells were harvested and seeded in 12-well plates at a density of 2 × 10^5^ cells/ml in DMEM/F12 and incubated overnight at 37 °C and 5% CO_2_. The culture medium was removed before adding Cx43AsODN:XP complexes in fresh DMEM/F12 for 24 h at 37 °C and 5% CO_2_. For hypoxic conditions, cells were incubated in hypoxic, acidic, ion-shifted ringers (HAIR) solution^37^ for 4 h at 37 °C and 5% CO_2._ The HAIR solution was removed before adding Cx43AsODN:XP complexes in fresh HAIR solution for 24 h at 37 °C and 5% CO_2_. Complex solutions were removed and cells were washed three times in ice-cold PBS. Unless otherwise mentioned all buffers were made according to recipes found in Abcam protocols. Cells were lysed with the radioimmunoprecipitation assay (RIPA) buffer and transferred to fresh microfuge tubes. Cell lysates were shaken at 4 °C for 20 min and centrifuged at 11.5 RCF. The supernatant was transferred into fresh precooled microfuge tubes and stored at −80 °C. Lysates were quantified using Biorad DC protein estimation kit (Bio-Rad Laboratories Inc.). Cell lysates (5–7.5 µg) were boiled in laemmli buffer with 10% beta-mercaptoethanol before loading them into lanes of a TGX mini protean gel (Bio-Rad Laboratories Inc.). The gel tank was filled with running buffer and the gel ran at 250 V for 30 min. The blot was transferred onto a methanol-activated polyvinylidene difluoride (PVDF) membrane (Bio-Rad Laboratories Inc.) in transfer buffer at 170 mA for 1 h. The membrane was blocked for 1 h in 1% bovine serum albumin/Tris-buffered saline (BSA/TBS) at room temperature and subsequently incubated overnight with 1:8,000 primary Cx43 (Cat.# C6219, Sigma-Aldrich) or 1:4,000 Glyceraldehyde 3-phosphate dehydrogenase (GAPDH) (Cat.# G9545, Sigma-Aldrich) antibody in 1% BSA/TBS at 4 °C. The membrane was then washed three times in 0.02% Tween20/TBS (TBS-T) before a 2 h incubation with 1:40,000 secondary anti-rabbit IgG horse radish peroxidase (HRP) antibody (Cat.# NA934V, GE Healthcare Life Sciences). The membrane was washed three times in 0.02% TBS-T, and incubated in chemiluminescent substrate as per instructions of the Pierce ECL substrate kit and bands were visualised using a Fujifilm LAS-4000 luminescence imager. Protein bands were quantified using ImageJ and statistical analysis was carried out using GraphPad Prism 7.

### Data Availability

All data generated or analysed during this study are included in this published article.

## Results and Discussion

### Complete complex formation and complex size using Zetasizer

**Figure 1:**
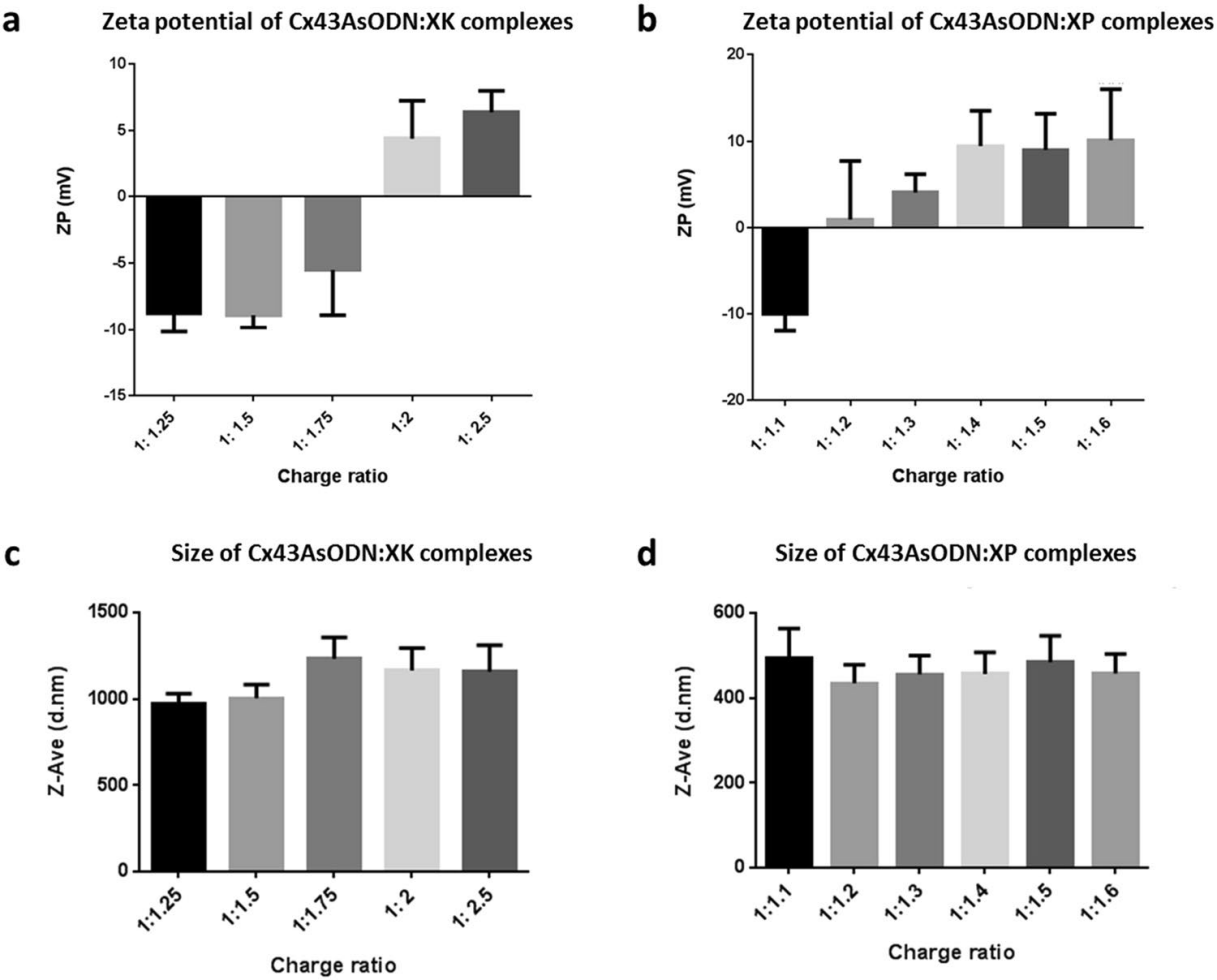
Zeta potential and size of Cx43AsODN:XK and Cx43AsODN:XP complexes at increasing charge ratios. (**a**) Zeta potential measurements showed that Cx43AsODN:XK complexes first reached a net positive charge at a ratio of 1:2. (**b**) Zeta potential measurements revealed that Cx43AsODN:XP complexes already reached a net positive charge at a ratio of 1:1.2. Size measurements of (**c**) Cx43AsODN:XK and (**d**) Cx43AsODN:XP revealed no significant differences in the overall size of the complexes formed at the various charge ratios tested; however, Cx43AsODN:XK complexes were at least double the size of Cx43AsODN:XP complexes. Data shown as mean + SD, n = 3 measurements per sample.

Cx43AsODN:XK and Cx43AsODN:XP complexes were formed by non-covalent electrostatic interactions as previously described^34–36^. The use of electrostatic interactions to bind DNA to protamine and KALA has been efficacious previously, therefore these were deemed good candidate peptides to use in combination with Xentry to form complexes with Cx43AsODN^35,38^. In addition, XK has also been used to successfully transport siRNA into mammalian cells^33^. A range of charge ratios was selected for both XK and XP in order to capture the point at which a net positive charge would be achieved indicating complete binding of all free Cx43AsODN (Fig. 1).

As seen in Fig. 1a, zeta potential measurements of Cx43AsODN:XK complexes showed that the shift from a net negative to a net positive charge occurred at a charge ratio of 1:2, while this occurred at a charge ratio of 1:1.2 for Cx43AsODN:XP complexes (Fig. 1b). The lower charge ratio required for XP to achieve complete complexation was somewhat expected as XP contains more than double the positive charges of XK. Size measurements of XK (Fig. 1c) and XP (Fig. 1d) complexes revealed that there was no significant change at any of the charge ratios tested. However, Cx43AsODN:XK complexes were larger than Cx43AsODN:XP complexes (1106.36 nm and 463.65 nm respectively). The difference in size can be contributed to a number of factors. The higher number of positively charged amino acids in XP (13 arginine residues) compared to XK (6 lysine residues) allows for a neutral complex with Cx43AsODN to be achieved with fewer XP than XK molecules thus resulting in an overall smaller complex size. In addition, arginine residues are known to result in more condensed complexes than lysine residues due to their intrinsic properties^36,39–41^. While XP is more positively charged, it also contains nine fewer amino acids than XK thus resulting in a further reduction in complex size. With all these factors combined it is not surprising that Cx43AsODN:XP complexes were smaller than Cx43AsODN:XK complexes. Overall, the larger size of Cx43AsODN:XK complexes was not a concern for cell uptake as Xentry has previously been used to transport large molecules such as antibodies into cells with high efficiency^33^.

### Complete complex formation by gel shift mobility assay

**Figure 2:**
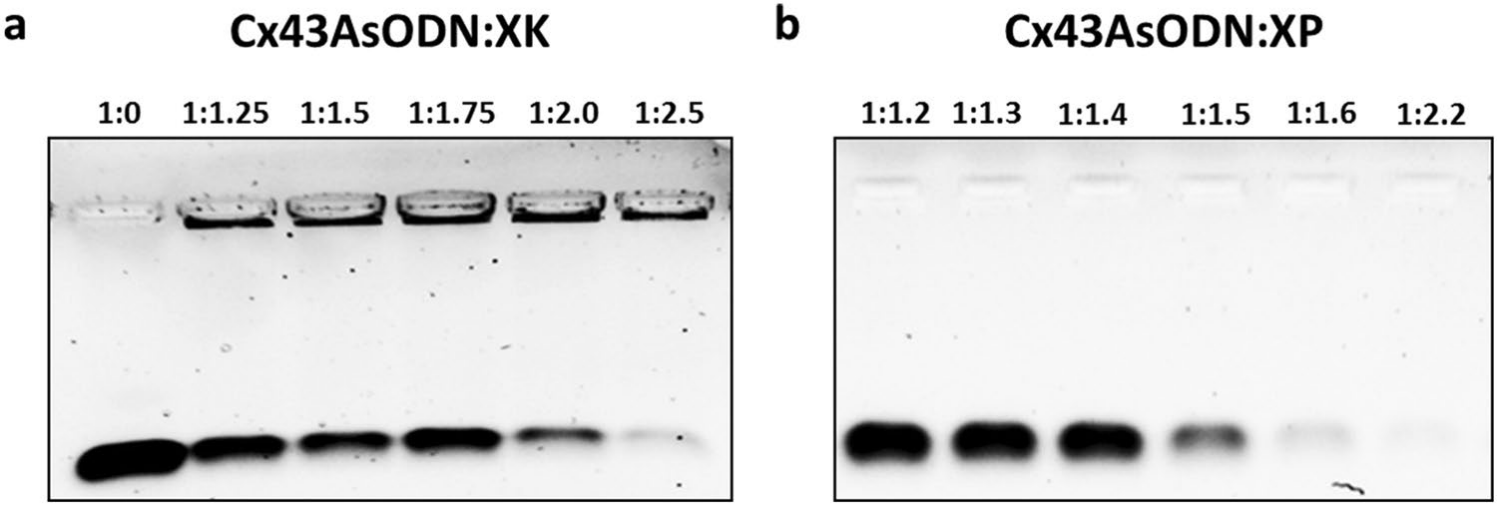
Gel shift mobility assay of Cx43AsODN:XK and Cx43AsODN:XP at increasing charge ratios. (**a**) Cx43AsODN:XK and (**b**) Cx43AsODN:XP complexes were loaded at increasing charge ratios into lanes of a 1% agarose gel. Gels were stained with GelRed™ nucleic acid stain and Cx43AsODN was visualised on a Bio-Rad Gel Doc EZ imaging system.

Oligonucleotides are easily broken down in the systemic circulation resulting in a short half-life within cells^20–23^. Therefore, any free Cx43AsODN not properly complexed to XP or XK may be easily broken down thus reducing efficacy. The gel shift mobility assay is a technique to study DNA-protein interactions^42^. Native unbound DNA migrates freely in the gel, while DNA bound to the peptide moves slowly or not at all through the gel depending on the availability of protein binding^43^. Therefore, complete complex formation of Cx43AsODN:XK and Cx43AsODN:XP was investigated by the gel shift mobility assay. Complexes were added into wells of a 1% agarose gel at increasing charge ratios. Uncomplexed negatively charged Cx43AsODN (Fig. 2a, left lane) migrated freely in the gel while positive complexes stayed in the loading wells as they were not drawn down by the current.

Complete complex formation of Cx43AsODN:XK (Fig. 2a) occurred at a charge ratio of 1:2.5 with no free Cx43AsODN migrating through the gel at this ratio. Cx43AsODN:XP (Fig. 2b) complex formation, on the other hand, already occurred at a lower charge ratio of 1:2.2 similar to zeta potential results. However, while zeta potential measurements suggested that an overall positive surface charge of these complexes can be achieved at much lower charge ratios (1:2 and 1:1.2 for Cx43AsODN:XK and Cx43AsODN:XP, respectively), the gel shift assay indicated that complete complex formation occurred at much higher charge ratios. This could be due to the solvent used as the complexes were formed in PBS for the gel shift mobility assay while complexes were formed in ultrapure water for zeta potential measurements. The type of medium used to form complexes has previously been shown to alter the charge; therefore, higher charge ratios were required for complete complex formation in PBS^44^. Previous literature has found a similar range of charge ratios (between 1:1.25 and 1:2.5) for DNA and KALA preventing DNA migration due to complex formation^35^. Interestingly, the DNA sample used in that study was composed of 28 nucleotides and thus similar to the number of nucleotides (30) used here. This suggested that the addition of Xentry to KALA did not affect its electrostatic binding potential and complete complex formation was achieved.

### Cx43AsODN:XK and Cx43AsODN:XP uptake into ARPE19 cells

ARPE-19 are immortalized human retinal pigment epithelium (RPE) cells which highly express Cx43 protein^45,46^. Using the charge ratios determined by the gel shift mobility assay (1:2.5 for Cx43AsODN:XK and 1.2.2 for Cx43AsODN:XP), XP and XK were mixed with 1 µM of Cx43AsODN to form complexes before treating ARPE-19 cells, with the delivery of 1 µM Cx43AsODN previously shown to knockdown Cx43 expression in uninjured spinal cords and acute skin wounds of rats^18,19,47^. Cx43AsODN used in the cellular uptake studies was tagged with Cyanine-3 (Cy3) allowing visualisation of Cx43AsODN uptake via red fluorescence.

**Figure 3:**
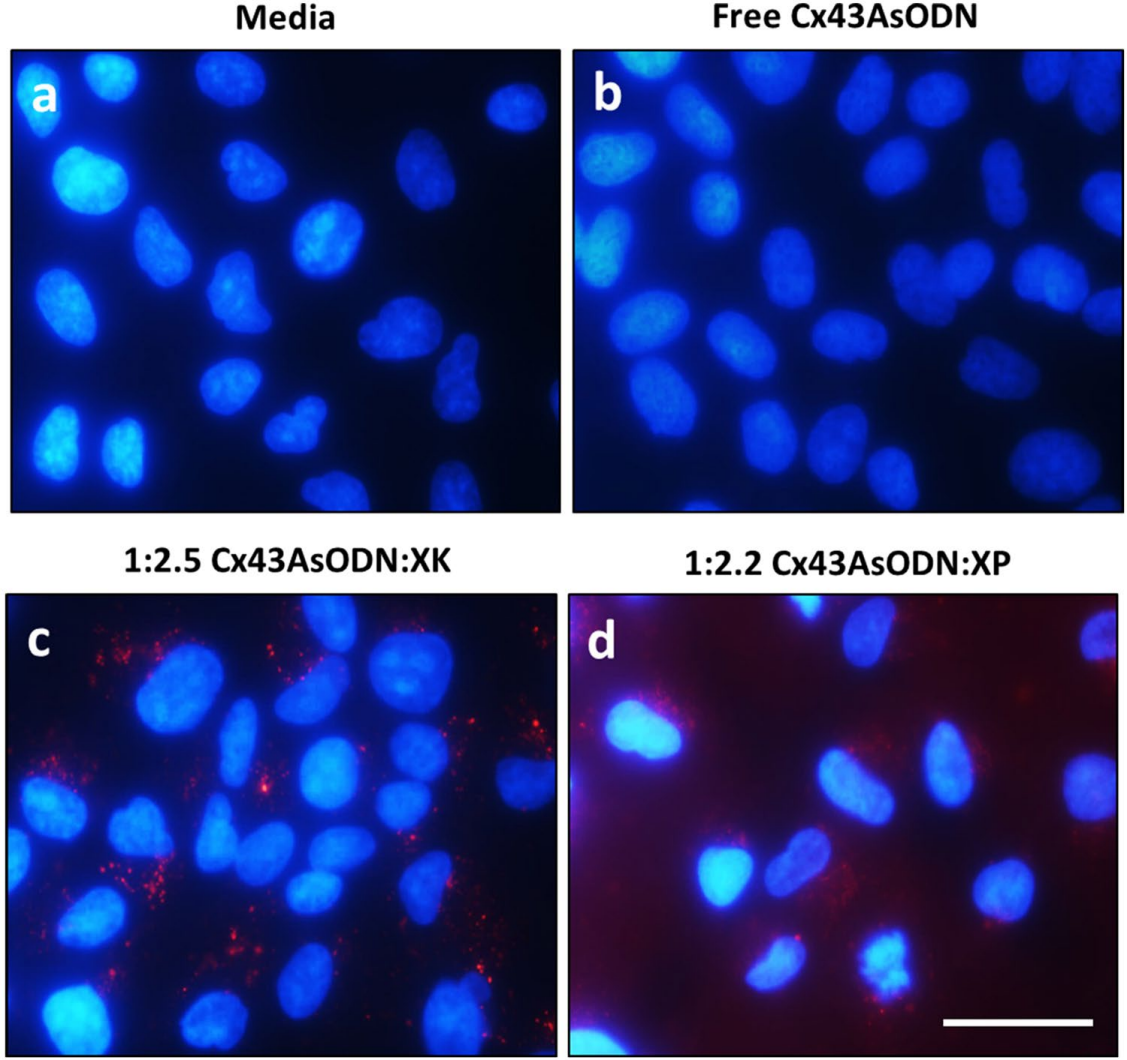
Cellular uptake of Cx43AsODN:XK and Cx43AsODN:XP by ARPE-19 cells. Cell nuclei were stained with DAPI (blue) and Cx43AsODN was Cy3-tagged (red). (**a**) Cells in media only, (**b**) treated with free Cx43AsODN-Cy3, (**c**) treated with 1:2.5 Cx43AsODN:XK and (**d**) treated with1:2.2 Cx43AsODN:XP (scale bar: 50 µm).

As seen in Fig. 3a, cells in media showed only blue fluorescence from DAPI staining the nucleus. No red Cy3 fluorescence was seen as Cx43AsODN was not applied to these cells. A similar result was observed when free Cx43AsODN (Fig. 3b) was applied directly onto the cells suggesting that Cx43AsODN on its own was unable to penetrate into ARPE-19 cells. This was likely due to breakdown of free Cx43AsODN in serum containing medium and/or poor uptake below the detection limit of the assay^20^. In contrast, application of Cx43AsODN:XK or Cx43AsODN:XP complexes (Fig. 3c,d, respectively) resulted in detectable red fluorescence inside the cell. Cx43AsODN:XK uptake resulted in a defined punctate circular appearance suggesting that Cx43AsODN:XK was localised within endosomes^32,48^. The inability of the cargo to escape the endosome would mean that Cx43AsODN would be unable to interfere with the mRNA in the cytoplasm to knockdown Cx43 protein levels. This was unexpected as KALA is known to aid endosomal escape by promoting endosomal leakage and improving transportation of oligonucleotides; however, this occurred at charge ratios of 1:10, DNA:KALA, which was much higher than what was used in our experiments^35^. Moreover, XK has previously been used for the delivery of double stranded siRNA against B-raf which resulted in successful apoptosis within melanoma cells^33^. Therefore, it is unlikely that the Xentry component hindered the endosomal escape of Cx43AsODN:XK. The inability to escape the endosome could be attributed to the overall larger size of the Cx43AsODN:XK complex as well as the overall lower net positive charge of lysine containing KALA. The intracellular distribution of Cx43AsODN:XP was very different to that of Cx43AsODN:XK. While some punctate circular vesicles were visible indicating endosomal uptake, the majority of the Cy3 fluorescence observed within the cells appeared dispersed resulting in a red fluorescent glow throughout the cells. This suggested that Cx43AsODN had successfully escaped the endosome into the cytoplasm where it could interfere with Cx43 mRNA as previously reported in multiple models^17,28,48–50^. Given its smaller size and better cellular uptake showing endosomal escape, only Cx43AsODN:XP was further investigated.

### Increased condensed cell appearance with increasing XP concentrations

Highly cationic peptides can cause cell morphology changes at higher concentrations^35,36,51^. Therefore, XP was applied to a monolayer of ARPE-19 cells at increasing concentrations and cells were assessed for any morphological changes.

**Figure 4:**
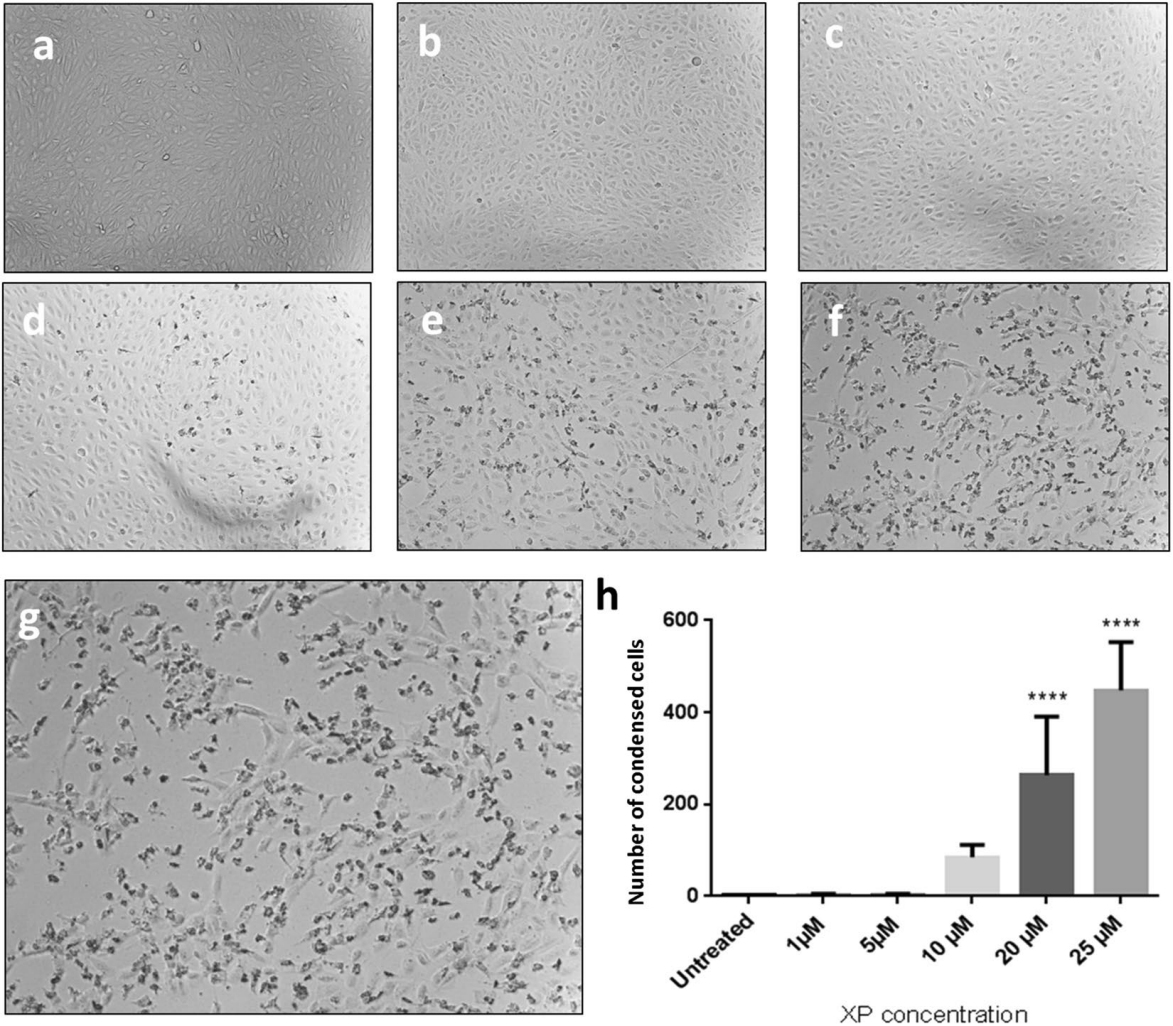
Increased cell condensation at increasing XP concentrations. ARPE-19 cells were either (**a**) left untreated or treated with (**b**) 1 µM, (**c**) 5 µM, (**d**) 10 µM, (**e**) 20 µM or (**f**) 25 µM of XP and cells were visualised using bright field light microscopy.(**g**) Enlarged image of cells treated with 25 µM of XP showing a large number of condensed cell bodies.(**h**) Five visual fields were captured per treatment and quantified using ImageJ. Statistical analysis was carried out using GraphPad Prism7. Data shown as mean + SD, n = 5, ****p < 0.0001).

Analysis of bright field images revealed that at 1 to 5 µM of XP (Fig. 4b,c) the cell morphology was unchanged and comparable to that of untreated cells (Fig. 4a). At 10 µM (Fig. 4d) some cell bodies began to condense and appeared round and dark compared to the normal elongated cells. At 20 and 25 µM, a dose dependant increase of condensed cells was seen (Fig. 4e,f) suggesting a change in cell morphology due to the highly positively charged peptides. The enlarged image of cells treated with 25 µM XP (Fig. 4g) clearly shows areas of condensed cells appearing dark and round while normal cells appear clear, flat and elongated. These abnormal cells were quantified and analysed by one-way ANOVA and Sidak’s post hoc test which revealed that there was no significant difference in the number of condensed cells from 1 to 5 µM of XP when compared to untreated cells (Fig. 4h). At 10 µM there was a slight increase in condensed cells, however, this was not significantly different from untreated cells. At 20 and 25 µM there was a significant increase (****p < 0.0001) in the number of condensed cell bodies suggesting that XP should only be used at concentrations below 10 µM to avoid changes in cell morphology. Xentry has previously been shown to be non-toxic in *in vitro* and *in vivo* assays^32,33^. While higher concentrations of protamine have previously been shown to cause lipid disruption in *in vitro* assays; protamine has successfully been administered intravenously into humans with minimal toxicity up to 600–800 mg^36,52^. Protamine is also a component of neutral protamine hagedorn (NPH) insulin^36^, a complex that increases the insulin half-life and duration of action. In addition, the complex is rendered non-antigenic enabling administration to diabetic patients throughout their lifespan^53^. The change in cell morphology could be due to the overall positive charge of XP alone. It is possible that when complexed with Cx43AsODN the overall change in cell morphology would be reduced due to the lower net positive charge.

### Cx43 protein expression post Cx43AsODN:XP uptake

The ability of Cx43AsODN:XP to knockdown Cx43 expression was analysed by Western blot. APRE-19 cells were treated with Cx43AsODN:XP and then lysed and probed for Cx43 and GAPDH as the loading control. To maintain a charge ratio of 1:2.2 Cx43AsODN:XP, ARPE-19 cells were treated with complexes of 5 µM Cx43AsODN and 25 µM XP. A higher concentration of Cx43AsODN was selected here to compensate for the higher cell density required to generate sufficient cell lysate for Western blot analysis.

**Figure 5:**
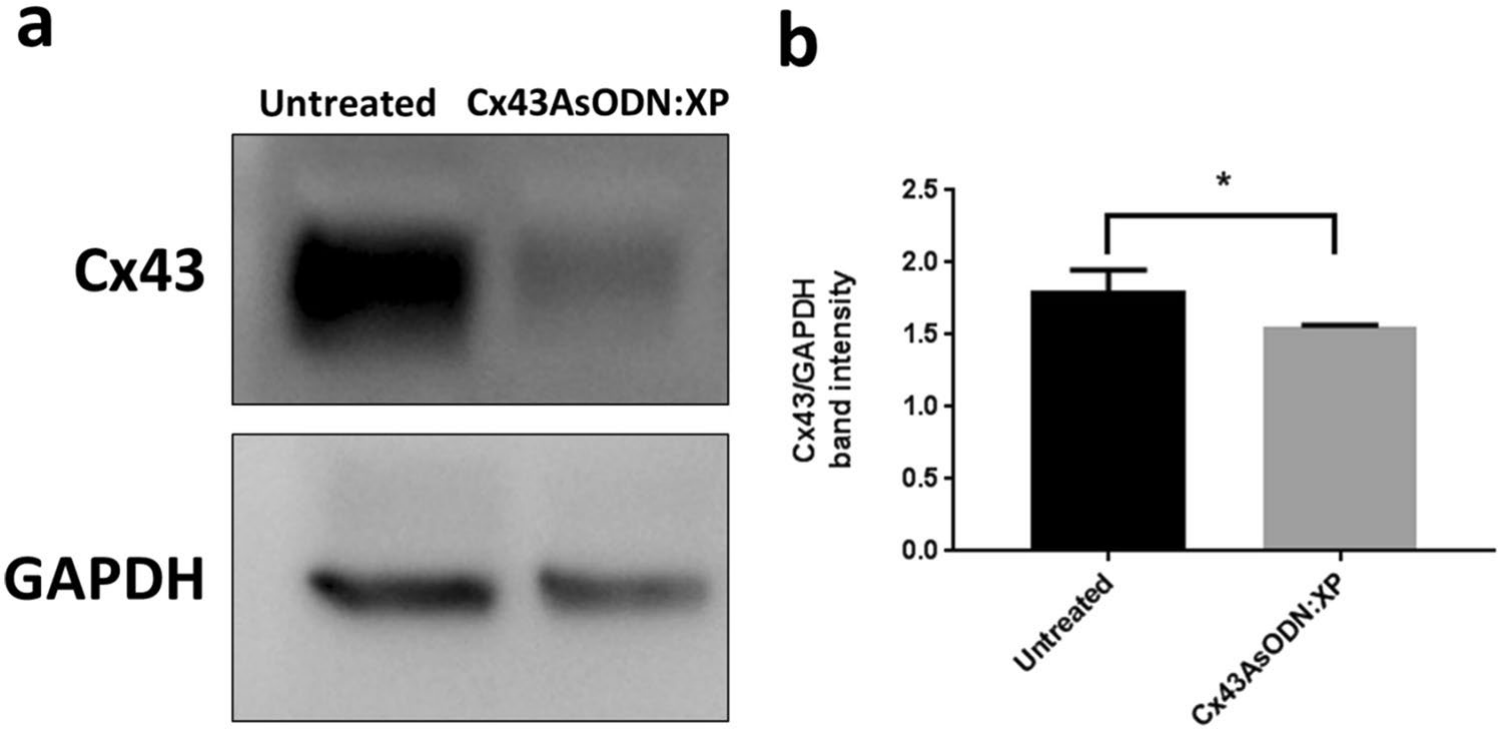
Cx43 knockdown post Cx43AsODN:XP treatment. (**a**) The figure is a cropped representation of the gel/blot obtained by detection of Cx43 and GAPDH. All bands shown were obtained and cropped from the same gel/blot. Full-length blots which include repeats are reported in Supplementary Fig. S1. ARPE-19 cells were treated with Cx43AsODN:XP at 5 µM Cx43AsODN and 25 µM XP to maintain a 1:2.2 charge ratio. Cell lysates from untreated cells in media alone showed elevated Cx43 band intensity compared to Cx43AsODN:XP treated cells. (**b**) Cx43 and GAPDH band intensities were quantified using ImageJ and graphed as Cx43 band intensity per GAPDH intensity to normalise loading. Unpaired T-test analysis revealed a significant difference in Cx43 expression per GAPDH protein levels between untreated and Cx43AsODN:XP treated lysates (*p < 0.05, mean + SD, n = 3).

Untreated cells in media alone showed high Cx43 protein levels in ARPE-19 cells as indicated by the formation of an intense band (Fig. 5a). In contrast, cells treated with Cx43AsODN:XP revealed a very faint Cx43 band suggesting that protein levels were much lower in these cells. Cx43 band intensities were quantified and normalised against GAPDH band intensities to account for the loading amount. Band intensity measurements (Fig. 5b) showed that there was significantly less Cx43 expressed in Cx43AsODN:XP treated cells compared to untreated cells (unpaired T-test, *p < 0.05, mean + SD, n = 3). These results showed that post uptake of Cx43AsODN:XP into ARPE-19 cells, Cx43AsODN remained functional and was in a bioavailable form allowing for interference with mRNA translation into Cx43 protein. This shows that XP can effectively deliver Cx43AsODN and reduce Cx43 expression in ARPE-19 cells. As mentioned previously the lack of Cx43 gap junction communication during recovery is detrimental to the cell^15^. The diminished Cx43 band seen in the Cx43AsODN:XP treated cells suggests that the Cx43AsODN effect was longer lasting than the half-life of the Cx43 protein and thus no new Cx43 was produced. Therefore, this concentration of Cx43AsODN delivered to the cell might be too high for therapeutic use as it could potentially affect normal gap junction communication^16,54–57^. While delivering high doses of Cx43AsODN could potentially be detrimental to the cell beyond the injury period, previous rat models have required much higher doses of Cx43AsODN to be administered for chronic conditions compared to acute injury^19,47,49,50^. Therefore, concentration specific formulations will need to be derived based on the acute or chronic nature of the disease being targeted.

### Cx43 protein expression under hypoxic conditions

Cx43 protein levels increase in response to hypoxic injury^58,59^. Therefore, Cx43AsODN:XP knockdown of Cx43 was investigated post hypoxic injury. ARPE-19 cells were subjected to hypoxic conditions by applying HAIR solution^37^. In these experiments the concentrations of XP and Cx43AsODN were reduced to 5 µM and 1 µM respectively (5-fold lower than in the above assay) while still maintaining the 1:2.2 Cx43AsODN:XP charge ratio. This was done to observe a transient knockdown in expression between normal and hypoxic cells without completely knocking down Cx43 protein in either condition which could result in significantly reduced gap junction coupling as a response.

**Figure 6:**
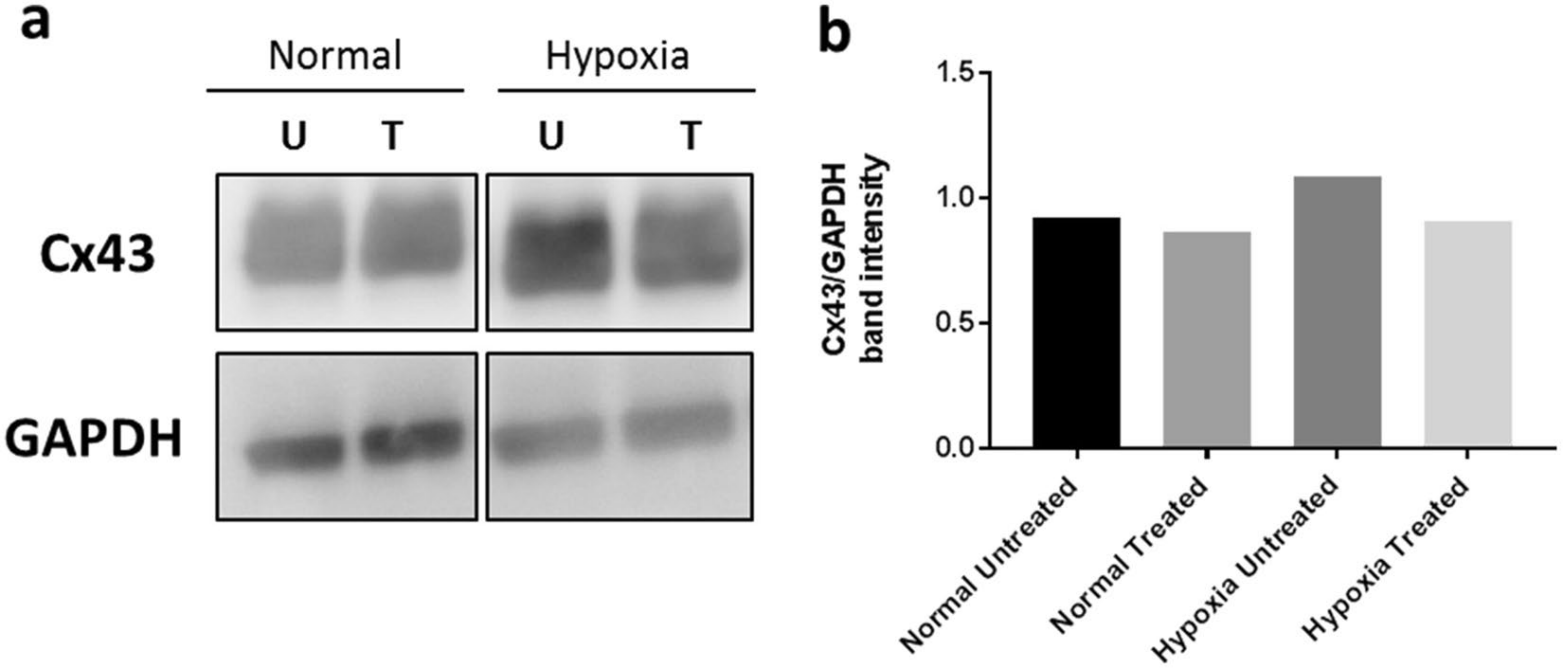
Western blot of normal and hypoxic cell lysates post Cx43AsODN:XP treatment. (**a**) The figure is a cropped representation of the gel/blot obtained by detection of Cx43 and GAPDH. All bands shown were obtained and cropped from the same gel/blot. Full-length blots are reported in Supplementary Fig. S2. (**a**) Cellular uptake of Cx43AsODN:XP was conducted in normal (left) and hypoxic (right) ARPE-19 cells. Cells were lysed and Cx43 and GAPDH expression was measured. (U) Untreated cells (T) Cx43AsODN:XP treated cells. (**b**) Cx43 and GAPDH band intensities were quantified using ImageJ and graphed as Cx43 band intensity per GAPDH intensity to normalise loading.

As seen in Fig. 6a, hypoxic cell lysates showed more intense Cx43 bands compared to lysates from normal cells. There was no difference in Cx43 expression between treated and untreated normal cell lysates. This is because under normal conditions there was no stimulus for higher Cx43 mRNA expression. At the same time the reduced concentration of Cx43AsODN applied to the cells only had a minimal effect and did not interfere with existing Cx43 protein. In contrast, the Cx43 band was more intense in untreated compared to Cx43AsODN:XP treated hypoxic cell lysates. This is because following hypoxic injury Cx43 mRNA expression increases^60^, thus delivered Cx43AsODN was able to reduce excessive Cx43 protein translation in hypoxic cells resulting in a less intense Cx43 band. The lowered concentration of Cx43AsODN:XP used in these experiments resulted in partial knockdown of Cx43 reducing expression back towards baseline levels in these hypoxic cells (Fig. 6b). Therefore, the reduced concentration allowed a knockdown in the expression of new Cx43 without affecting baseline Cx43 levels which would be required for normal cell function. These results confirmed that Cx43AsODN:XP successfully delivered its cargo inside the cells to knockdown Cx43 protein levels during hypoxic injury.

## Conclusion

We have shown here that two cationic peptides, XK and XP, were able to successfully transport Cx43AsODN inside ARPE-19 cells after complex formation. XP formed smaller complexes at lower charge ratios compared to XK resulting in more efficient cell uptake and endosomal escape. Cx43AsODN:XP intracellular delivery achieved Cx43 protein knockdown in ARPE-19 cells demonstrating that the cargo, Cx43AsODN, was delivered in a bioavailable and functional form. It is important to consider the concentration of Cx43AsODN delivered to the cell as complete protein knockdown could be detrimental to cell function. Therefore, it was encouraging that the reduced concentration of Cx43AsODN:XP was also able to return Cx43 expression back towards baseline levels under hypoxic conditions. This study highlights the potential of XP and XK to deliver functional oligonucleotides into cells. Future studies will evaluate the stability and systemic delivery of these complexes in animal disease models.

## Electronic supplementary material

Figures S1 and S2
