# Supplementary material for: Intracellular oligonucleotide delivery using the cell penetrating peptide Xentry
Source: Sci Rep. 2018 Jul 26;8:11256. doi: 10.1038/s41598-018-29556-7 (PMC6062516; doi:10.1038/s41598-018-29556-7)
Supplement: Supplementary file 1 — Figures S1 and S2 [file 41598_2018_29556_MOESM1_ESM.pdf]

Intracellular oligonucleotide delivery using the cell penetrating peptide Xentry

Authors:

Frazer P. Coutinho[1, 2], Colin R. Green[2], Ilva D. Rupenthal[1, 2*]

Affiliations:

[1] Buchanan Ocular Therapeutics Unit, Department of Ophthalmology and the New Zealand National Eye Centre, University of Auckland, New Zealand
[2] Department of Ophthalmology and the New Zealand National Eye Centre, University of Auckland, New Zealand

***Corresponding Author:**

Dr Ilva D. Rupenthal
Tel.: +64 9 923 6386
E-mail address: i.rupenthal@auckland.ac.nz

This file includes:

1. Supplementary Figure S1

2. Supplementary Figure S2

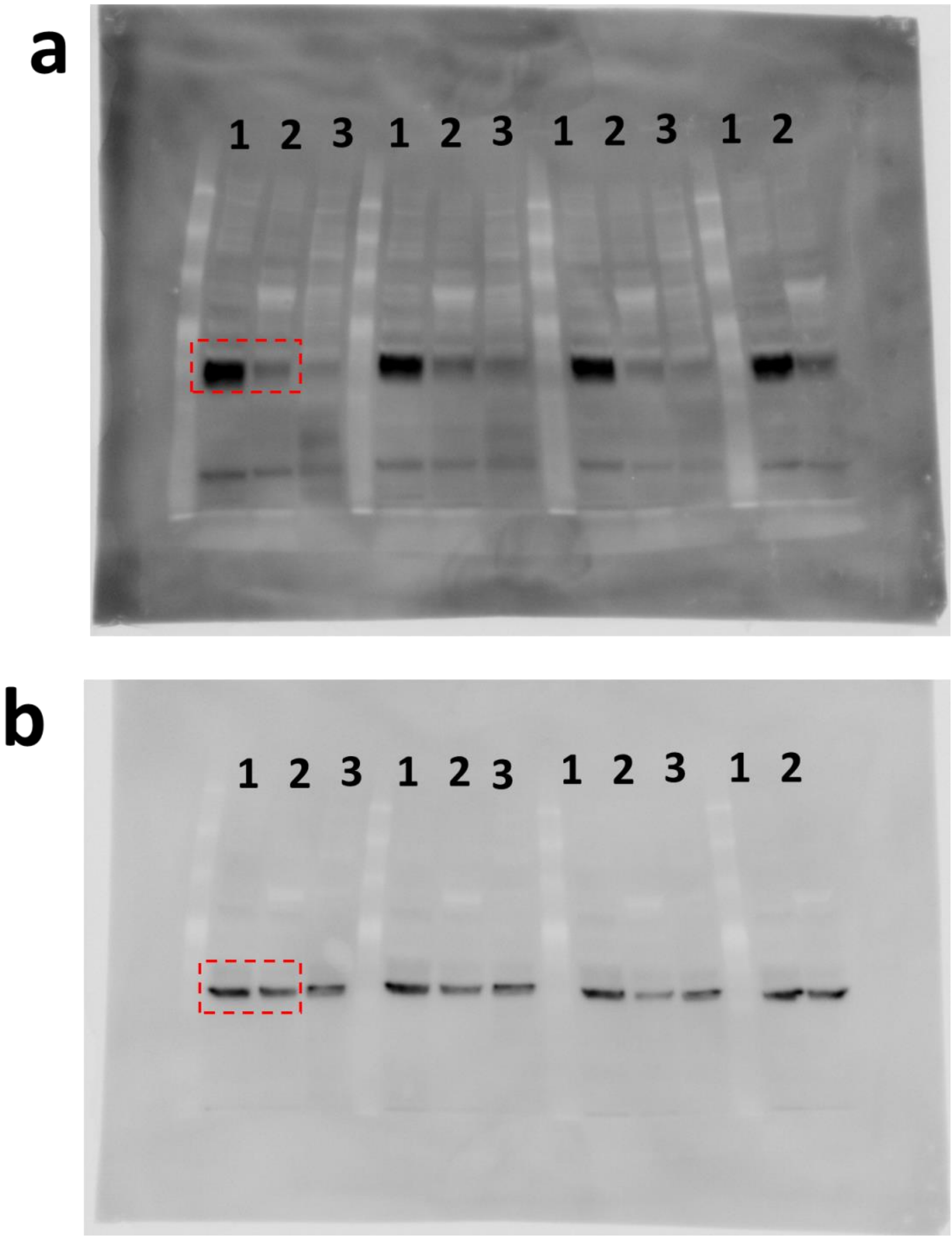

Supplementary Figure S1. Full length blots of Figure 5. (a) Cx43 and **(b)** GAPDH detection from **(1)** Untreated cell lysates **(2)** Cx43AsODN:XP treated lysates and **(3)** HeLa cell lysates as control**.** Red dotted lines indicate the cropped representative image used in the article.

a

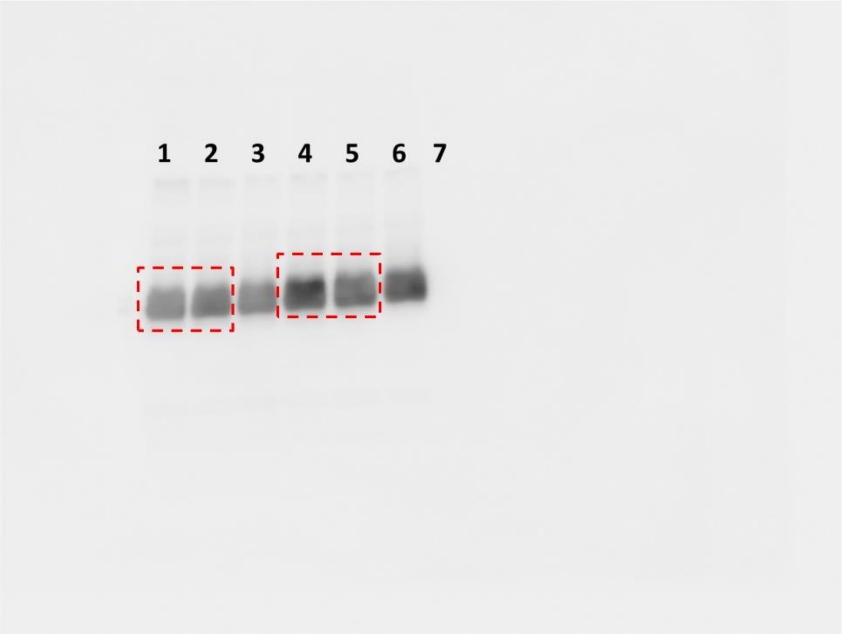

b

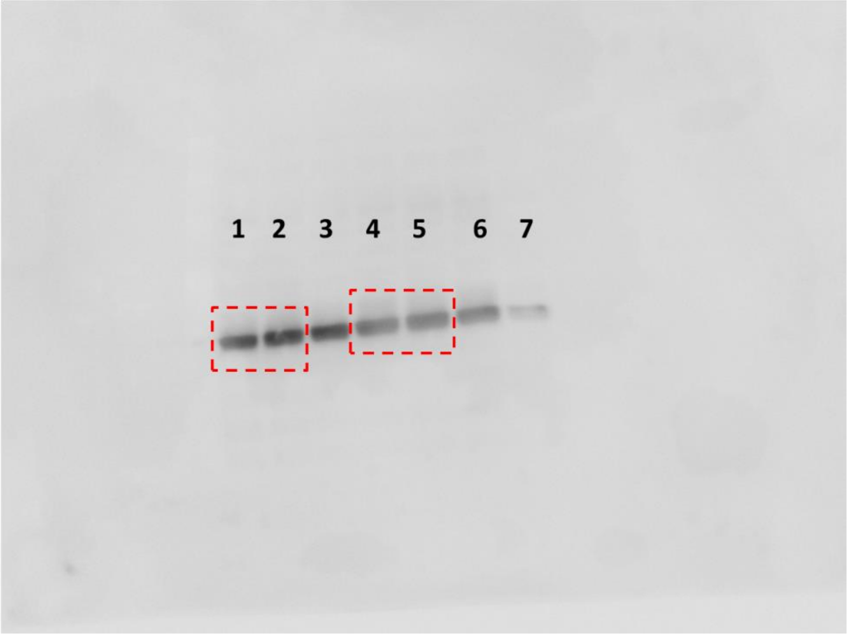

Supplementary Figure S2. Full length blots of Figure 6. (a) Cx43 and **(b)** GAPDH detection from **(1)** Untreated lysates of cells under normal conditions, **(2)** Cx43AsODN:XP treated lysates of cells under normal conditions, **(3)** Cx43AsODN:Oligofectamine treated lysates of cells under normal conditions, **(4)** Untreated lysates of cells under hypoxic conditions, **(5)** Cx43AsODN:XP treated lysates of cells under hypoxic conditions, **(6)** Cx43AsODN:Oligofectamine treated lysates of cells under hypoxic conditions as control and **(7)** HeLa cell lysates as control. Red dotted lines indicate the cropped representative image used in the article.
